# Free-Standing Undoped ZnO Microtubes with Rich and Stable Shallow Acceptors

**DOI:** 10.1038/srep27341

**Published:** 2016-06-06

**Authors:** Qiang Wang, Yinzhou Yan, Yong Zeng, Yue Lu, Liang Chen, Yijian Jiang

**Affiliations:** 1 Institute of Laser Engineering, Beijing University of Technology, Beijing 100124, China; 2 Beijing Engineering Research Center of 3D Printing for Digital Medical Health, Beijing University of Technology, Beijing 100124, China; 3 Institute of Microstructure and Property of Advanced Materials, Beijing University of Technology, Beijing 100124, China; 4 College of Applied Sciences, Beijing University of Technology, Beijing 100124, China

## Abstract

Fabrication of reliable large-sized *p*-ZnO is a major challenge to realise ZnO-based electronic device applications. Here we report a novel technique to grow high-quality free-standing undoped acceptor-rich ZnO (A-ZnO) microtubes with dimensions of ~100 μm (in diameter) × 5 mm (in length) by optical vapour supersaturated precipitation. The A-ZnO exhibits long lifetimes (>1 year) against compensation/lattice-relaxation and the stable shallow acceptors with binding energy of ~127 meV are confirmed from Zn vacancies. The A-ZnO provides a possibility for a mimetic *p-n* homojunction diode with *n*^+^-ZnO:Sn. The high concentrations of holes in A-ZnO and electrons in *n*^+^-ZnO make the dual diffusion possible to form a depletion layer. The diode threshold voltage, turn-on voltage, reverse saturated current and reverse breakdown voltage are 0.72 V, 1.90 V, <10 μA and >15 V, respectively. The A-ZnO also demonstrates quenching-free donor-acceptor-pairs (DAP) emission located in 390–414 nm with temperature of 270–470 K. Combining the temperature-dependent DAP violet emission with native green emission, the visible luminescence of A-ZnO microtube can be modulated in a wide region of colour space across white light. The present work opens up new opportunities to achieve ZnO with rich and stable acceptors instead of *p*-ZnO for a variety of potential a*p*plications.

## Introduction

ZnO has attracted considerable attention over past decades due to its wide potential applications, *e.g.* photocatalytic, antibacterial, piezoelectric, room-temperature ferromagnetic, magneto-optic, chemical-sensing, doping-depended electrically conductive properties, *etc*^1^,^2^,^3^,^4^,^5^. As a representative of the 3^rd^ generation semiconductors, the wide band-gap (~3.37 eV) with a large exciton binding energy (~60 meV) and biexciton binding energies (on the order of 25 meV thermal energy) at room temperature make ZnO attractive for low-cost and high-efficiency LEDs and lasers near the UV band^6^,^7^. Unfortunately, a major challenge for development of ZnO-based devices is to stabilise high concentrations of hole-carriers at room temperature. It is well known that ZnO grown by conventional techniques frequently possesses high levels of *n*-type conductivity that is attributed to the native defects (*e.g.* oxygen vacancies V_O_ and zinc interstitial Zn_i_) and hydrogen incorporation. These defects may cause *self-compensation* during *p*-type doping, *i.e.* the certain native donor defects spontaneously formed to compensate the doped acceptors^8^,^9^,^10^. Numerous efforts on *p*-type dopants (*e.g.* N, Sb, As, N-In, *etc.*) have been performed in the past decades^11^,^12^,^13^,^14^,^15^. Unfortunately, the self-compensation and lattice relaxation from native defects still block to achieve reliable *p*-type ZnO bulk with long lifetimes. Several studies have revealed that the non-equilibrium processes in fabrication (*e.g.* CVD, MBE, laser irradiation, *etc.*) are beneficial to hold the high concentrations of hole-carriers and suppress the self-compensation in films and nanowires^16^,^17^,^18^,^19^,^20^. However, these methods are unsuitable for large-sized bulk and most importantly, the non-equilibrium-generated structures could be unstable under the significant change of ambience (*e.g.* high temperature). The non-equilibrium structures would transform to equilibrium ones and therefore reduce the concentration of hole-carriers^18^,^21^,^22^. Although the native defects in ZnO have been sophisticatedly investigated and understood^23^, the stable and reliable acceptors in ZnO bulk are rarely achieved experimentally. Undoubtedly, the stable undoped acceptor-rich ZnO (A-ZnO) is the base of reliable doped *p*-ZnO for *p-n* homojunction possible. Therefore, it is essential to explore the feasibility of large-sized undoped A-ZnO growth with high stability and reproducibility, as well as the corresponding electrical and optical properties. Theoretical studies have predicted that Zn vacancies (V_Zn_) are the dominant native acceptor defects with binding energy of 100–200 meV above the valence-band maximum (VBM)^23^,^24^,^25^,^26^. However, little experimental evidence of V_Zn_-dominated A-ZnO bulk are presented so far.

In this work, reliable free-standing undoped A-ZnO microtubes with sub-centimetre sizes were efficiently fabricated for the first time. The ZnO micro-/nano-tubes/rods have been widely studied due to its tuneable optical and electronic properties with novel architectures^27^,^28^,^29^,^30^. Micro-sized ZnO robs/tubes are more attractive in micro-optics as Fabry-Perot and optical whispering gallery cavities^27^,^30^. The present work provided strong evidence of V_Zn_ acting as stable shallow acceptors with high concentration in ZnO microtubes grown by a quasi-equilibrium process. It opens up new opportunities for developing novel types of reliable large-sized ZnO-based devices and further understanding the native acceptor defects in ZnO. The unique temperature-dependent photoluminescence (PL) by donor-acceptor-pairs (DAP) transition in A-ZnO was also observed, for the first time. As prospective, the undoped A-ZnO microtubes can be candidates for multi-wavelength light sources and thermosensors.

### Fabrication of undoped acceptor-rich ZnO (A-ZnO) microtubes

**Figure 1:**
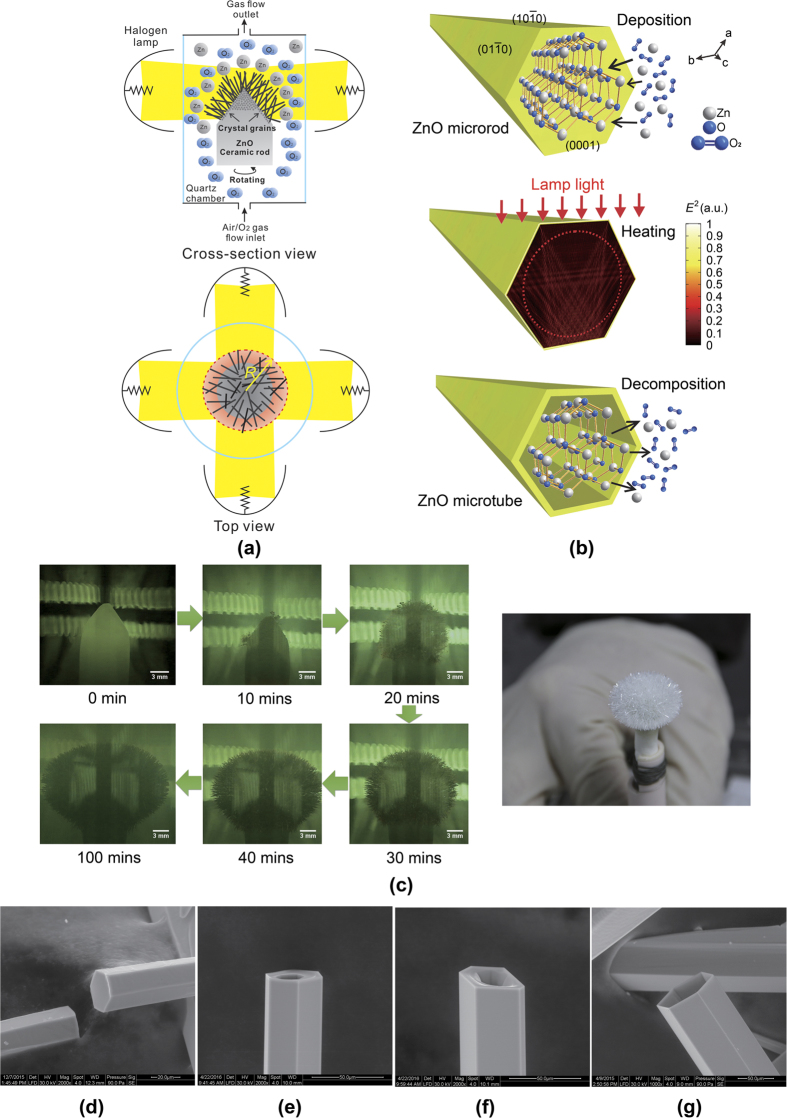
Growth of free-standing undoped A-ZnO microtubes. (**a**) Schematic of A-ZnO microrods/tubes growth by OVSP (as red region indicated in the top-view panel). (**b**) Mechanism of a single A-ZnO microrod growth by OVSP and microtube formation by thermal re-decomposition. The red dash circle indicates the decomposition area due to optical heating in an A-ZnO microrod. (**d**) Growing process of flower-like A-ZnO microtubes from a ceramic precursor rod. (**d**–**g**) The morphologies of hexagonal A-ZnO microrod and microtube growing at (**d**) 40 mins, (**e**) 60 mins, (**f**) 80 mins, and (**g**) 100 mins.

Figure 1a illustrates the fabrication process of A-ZnO microtubes. The growth was carried out in a cylindrical quartz chamber with controllable gas atmospheres. Light from four halogen lamps was focused at the chamber centre by ellipsoidal mirrors in order to form a homogenised optical heating zone, where a ZnO ceramic precursor rod was positioned and rotated for uniformed heating. Figure 1b shows the two stages of A-ZnO microtubes growing from the ceramic precursor rod: (i) microrods growth and (ii) microtubes formation. In stage (i), the ceramic precursor rod was heated by the focused light and then decomposed into Zn(g) and O_2_ at ~2000 °C. The concentration of Zn(g) dramatically increased with temperature elevating. When Zn(g) was supersaturated in the gas atmosphere (typically within 10 mins), Zn atoms would deposit on the precursor rod and meanwhile react with O_2_ to form ZnO(s), as the top panel in Fig. 1b. The micro-sized crystal grains in the precursor rod served as nucleation seeds at the beginning growth of ZnO microrods. According to the anisotropic structure of ZnO, the growth of microrods should point to the crystallographic direction of [0001]^31^,^32^. Oxygen-rich atmosphere was found to be a vital condition for microrod formation, in which additional O_2_ was supplied from the gas flow inlet into the chamber. The microrods continually grew within 40 mins and then the growth rate was reduced (as Fig. 1c) due to the concentration of Zn(g) *C*_*Zn*(*g*)_ ∝ 1/*R*^3^ under the balanced decomposition process, where *R* is the distance from the top centre of precursor rod to the tips of microrods. The growth stopped when *C*_*Zn*(*g*)_ was down to a threshold where the rate of deposition was equal to that of thermal decomposition. The growth process is based on optical vapour supersaturated precipitation (OVSP) in a uniformed temperature field. It is obviously different from previous studies using temperature gradients as the growth driving force^18^,^19^,^33^. The thermal-fluctuation-induced lattice mismatching, which is generally considered to be the root of acceptor instability^22^, can be dramatically reduced by OVSP. Moreover, the quasi-equilibrium growing process in oxygen-rich ambience guarantees formation energy of V_zn_ is lower than V_O_, in order to supress V_O_ and encourage V_Zn_ formation in ZnO^8^,^23^. Therefore, extensive V_Zn_ acceptors can be formed for A-ZnO. Figure 1d shows the morphology of an A-ZnO microrod growing within 40 mins. The hexagonal geometry validates the wurtzite-type structure of the ZnO microrod grown along *c*-axis, *i.e.* [0001]. In stage (ii), the incident light was focused by the hexagonal ZnO microrod (as the middle panel in Fig. 1b by numerical simulation) and the microrod centre was heated during precursor rod rotation, by which the ZnO at the microrod centre was re-decomposed from the metastable polar plane (0001)^34^ whereas the facets were remained due to the balance of thermal decomposition and vapour supersaturated deposition. The thin-wall microtubes were hence formed as shown in the bottom panel of Fig. 1b. Figure 1e,f demonstrate the thermal re-decomposition from the microrod centre during growing at 60 mins and 80 mins, respectively. The growing process of microtubes generally finished within 100 mins. Figure 1g demonstrates the morphology of a finish microtube, of which the dimensions are ~100 μm ×5 mm with the wall thickness of ~2 μm. It can be clearly seen that the prefect surface finish and free of contaminations were achieved. Figure 1c shows thousands of the free-standing microtubes growing up simultaneously by OVSP and forming a flower-like architecture on the precursor rod.

### Microstructures of undoped A-ZnO microtubes

**Figure 2:**
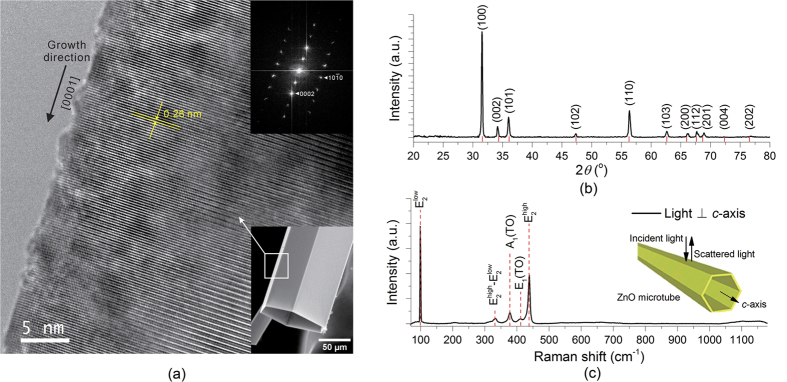
Microstructure of undoped A-ZnO microtubes. (**a**) HRTEM micrograph and corresponding FFT from the rectangular region of a single A-ZnO microtube (as marked in the inset). (**b**) XRD pattern of powdered A-ZnO microtubes. (**c**) Unpolarised Raman spectrum for backscattering perpendicular to the *c*-axis of the A-ZnO microtube.

Figure 2a shows the microstructure and corresponding fast Fourier transform (FFT) from the rectangular region of a single A-ZnO microtube by high-resolution transmission electron microscopy (HRTEM). The microtube possesses a single-crystalline structure with the growth direction of [0001] (*i.e. c*-axis orientation) along the microtube axis. The lattice fringes of 0.26 nm correspond to crystallographic plane of (0002) in wurtzite-type ZnO. There are no stacking faults or dislocations in the microtube. The crystalline phase and purity of the as-grown A-ZnO microtubes were examined by X-ray diffraction (XRD) and unpolarised Raman spectroscopy at room temperature, as Fig. 2b,c. As indexed in the XRD pattern, the phase of A-ZnO microtube is close to the standard wurtzite-type structure in space group *P6*_*3*_*mc*, where the lattice constant *a* = 3.267 Å and *c* = 5.219 Å. The five Raman active modes indicate the unpolarised Raman backscattering perpendicular to the *c*-axis of ZnO^35^. The growth direction of ZnO microtube is further validated along [0001] (as the inset in Fig. 2c). The HRTEM, XRD and Raman spectra confirm the pure-phase and wurtzite-type of A-ZnO single-crystal microtubes grown by OVSP.

### PL characteristics of undoped A-ZnO microtubes

**Figure 3:**
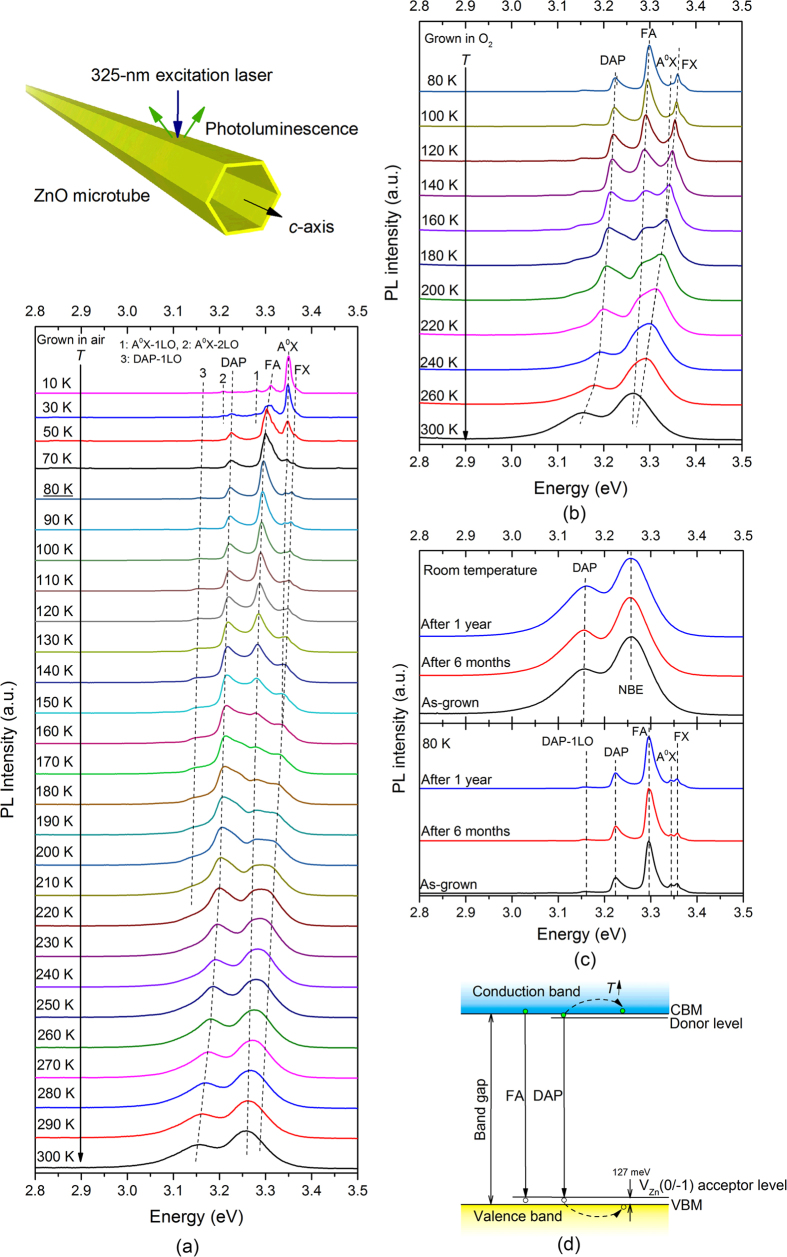
PL spectra of an undoped A-ZnO microtube. (**a**,**b**) Temperature-dependent PL spectra of an undoped A-ZnO microtube grown by OVSP in (**a**) air and (**b**) O_2_. (**c**) Aging effect of an undoped A-ZnO microtube. (**d**) Energy-level diagram of an undoped A-ZnO microtube with high concentration of V_Zn_.

Figure 3a shows the temperature-dependent PL spectra of a single undoped A-ZnO microtube. At 10 K, the free exciton (FX) transition at 3.373 eV is significantly suppressed due to high intensity of the dominant peak at 3.350 eV related to neutral acceptor-bound exciton (A^0^X) transition^36^,^37^. It is evidence of substantial acceptors existing in the microtube. The peaks at 3.280 eV and 3.210 eV are longitudinal optical (LO) phonon replicas (A^0^X-*m*LO, *m* = 1 and 2) of the A^0^X transition, with an energy separation of ~70 meV. The observed higher-order LO phonon replicas confirm the good crystalline quality and high purity of the A-ZnO microtube^36^. Furthermore, the peak at 3.310 eV becomes dominant when the temperature is in the range of 50–130 K. According to previous studies, the photon energy of ~3.310 eV is attributed to the free-electron-to-neutral-acceptor (FA) transition strongly related to the shallow acceptors^12^,^14^,^38^,^39^. The acceptor binding energy, *E*_*A*_, can therefore be estimated by^40^


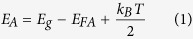


where *E*_*g*_ is the ZnO bandgap at low temperature (*E*_*g*_ = 3.437 eV^36^), *E*_*FA*_ is the FA transition energy, *k*_*B*_ is Boltzmann’s constant, and *T* is the temperature. The acceptor binding energy was calculated to be 127 meV, in good agreement with the theoretical V_Zn_ (0/−1 transition state) acceptor level of 0.1–0.2 eV above the VBM identified by the first-principles^23^,^24^,^25^,^26^. The hypothesis of acceptor-related emission from V_Zn_ with 3.310 eV is hence experimentally validated by the A-ZnO microtube. The transition energy of DAP (*E*_*DAP*_) located at 3.227 eV with the phonon replicas at 3.157 eV can also been observed in Fig. 3a^12^. The transition energy of DAP shifts toward lower energy of ~3.156 eV with the bandgap narrowed by temperature increasing (≤300 K). The appeared DAP emission peak at room temperature further confirms the presentence of abundant V_Zn_-related acceptors in the microtube. Figure 3b demonstrates the temperature-dependent PL spectra of ZnO microtubes grown in O_2_ down to 80 K, of which the positions of emission peaks (DAP, FA, A^0^X and FX) are same with those grown in air. Although the relative intensities of these peaks are slightly different, the high intensity of FA peak and appeared DAP/A^0^X peaks at low temperature indicate abundant acceptors existing in ZnO microtubes in oxygen growth as well. However, the microtubes cannot be grown in oxygen-deficient (*e.g.* Ar or N_2_) ambience in the control experiments. The oxygen-rich atmosphere is therefore a vital condition to grow undoped A-ZnO microtubes with high concentration of V_Zn_. It has been acknowledged that the *p*-type instability is the major challenge to ZnO-based devices, where the *p*-type conductivity is prone to convert into *n*-type over time due to *self-compensation* and lattice-relaxation in *p*-ZnO. The phenomenon has been widely witnessed in p-ZnO by non-equilibrium growth^18^,^21^,^22^. The aging test, as shown in Fig. 3c, exhibits the long lifetime of V_Zn_-dominated acceptor level (related to DAP, FA and A^0^X) in ZnO microtubes grown by quasi-equilibrium OVSP, where no obvious change in peaks is observed in the temperature-dependent PL spectra with various periods of as-grown, 6 months and 1 year. The V_Zn_-dominated acceptor level in the undoped A-ZnO microtube is therefore validated with high stability against compensation/relaxation. According to the PL analysis, the major energy levels of the undoped A-ZnO microtube grown by OVSP can be identified as shown in Fig. 3d.

### X-ray photoelectron spectroscopy (XPS) analysis of undoped A-ZnO microtubes

**Figure 4:**
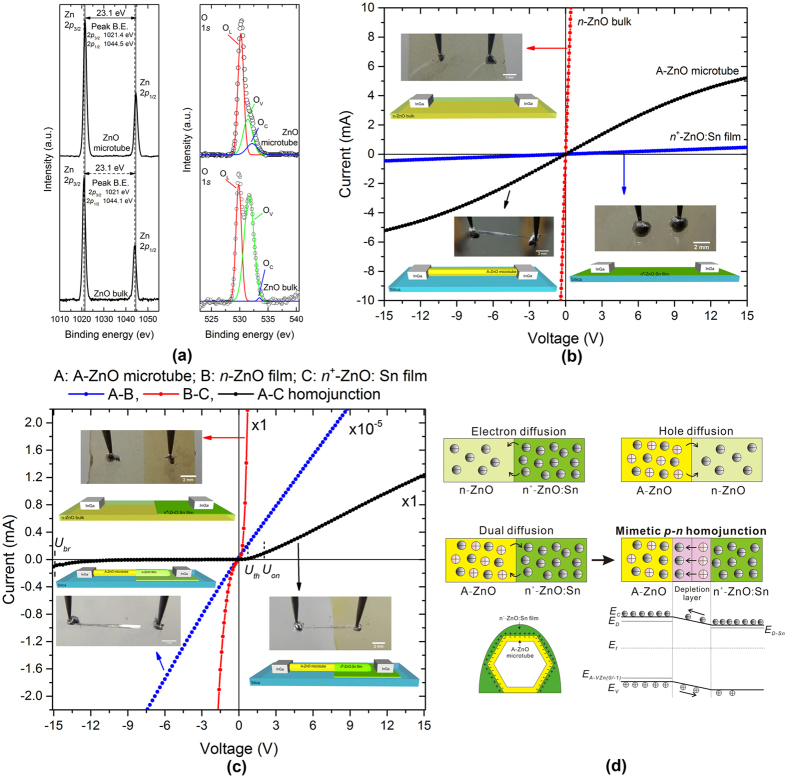
Electrical characteristics of undoped A-ZnO microtubes. (**a**) XPS spectra of Zn 2*p* and O 1*s* of undoped A-ZnO microtubes and undoped *n*-type ZnO single-crystal bulk. (**b**) *I-V* characteristics of a single undoped A-ZnO microtube, undoped *n*-ZnO bulk and *n*^+^-ZnO:Sn film. (**c**) *I-V* characteristics of A-ZnO-based mimetic *p-n* homojunction diode and *n-n*^+^ ZnO junction. (**d**) Schematics of *n-n*^+^ ZnO homojunction, A-ZnO-based mimetic *p-n* homojunction and the corresponding energy-level diagram.

XPS was further employed to confirm the origin of hole-carriers introduced in A-ZnO microtubes. Figure 4a shows the XPS spectra of as-grown undoped A-ZnO microtubes in this work and standard undoped *n*-ZnO single-crystal bulk from a commercial company. It can be seen that the binding energies of Zn 2*p* and O 1*s* are obviously different from the A-ZnO microtubes to the *n*-ZnO bulk. The two peaks at ~1021 eV and ~1045 eV with 23.1 eV separation are ascribed to Zn 2*p*^3/2^ and Zn 2*p*^1/2^, respectively, indicating the presentence of zinc atom in the form of Zn^2+^ 41. In the left panel of Fig. 4a, the Zn 2*p* binding energies of A-ZnO microtubes are slightly higher than the *n*-ZnO bulk ~0.4 eV. It means the high concentration of V_Zn_ and low concentration of V_O_ formed in the microtubes with the oxygen-rich atmosphere in growth, which lowered the formation energy of V_Zn_ (as hole-carriers) and increased the formation energy of V_O_ (as electron-carriers)^8^,^23^,^42^,^43^. The O 1*s* spectra in the right panel of Fig. 4a were fitted by three Gaussian peaks at 530.4 ± 0.15 eV (O_L),_ 531.8 ± 0.2 eV (O_V_), and 532.8 ± 0.15 eV (O_C_), associated with O^2−^ ions in the lattice (Zn-O bond), oxygen vacancies/defects, and chemisorbed/dissociated oxygen spices or OH^−^ on the surface of ZnO, respectively^44^,^45^. The ratio of peak intensity of O_V_ to O_L_ further confirms the low concentration of V_O_, by which the number of electron-carriers were reduced. Therefore, the concentrations of hole- and electron-carriers should be comparable in the A-ZnO microtubes.

### Electrical property of a single undoped A-ZnO microtube

In order to distinguish the electrical properties of undoped A-ZnO from undoped *n*-ZnO and doped *n*^+^-ZnO, *I-V* characteristics of a single A-ZnO microtube, undoped *n*-ZnO bulk and *n*^+^-ZnO:Sn film were acquired as shown in Fig. 4b. It should be noted that the variable current values of three materials were resulted from the different structural types, *i.e.* microtube, bulk and film. The A-ZnO microtube demonstrates a typical undoped semiconductor behaviour, of which the existed saturation voltage (>15 V) reveals low concentrations of carriers compared with undoped *n*-ZnO bulk and *n*^+^-ZnO:Sn film. The electrical resistivity of a single microtube is ~2.31 × 10^−2 ^Ω·cm (see Methods). In addition, it was found that In/Ga electrodes were suitable to achieve ohmic contacts for all types of ZnO samples, as shown in Fig. 4b. Hence, they were employed onto the below-mentioned homojunction for further investigation of A-ZnO-based diode.

### A-ZnO-based mimetic *p-n* homojunction diode

The undoped A-ZnO microtube was partially deposited by *n*^+^-ZnO:Sn film to form a homojunction of A-ZnO – *n*^+^-ZnO:Sn. The undoped *n*-ZnO – *n*^+^-ZnO:Sn and A-ZnO – undoped *n*-ZnO homojunctions were also fabricated for comparison. As shown in Fig. 4c, the A-ZnO – *n*^+^-ZnO:Sn homojunction demonstrates a diode-like behaviour at room temperature, where the rectification ratio is ~109 at 5 V. The threshold voltage (*U*_*th*_), turn-on voltage (*U*_*on*_), reverse breakdown voltage (*U*_*br*_), and reverse saturation current of the homojunction diode are 0.7 V, 2.1 V, >15 V, and <10 μA, respectively. The mimetic *p-n* homojunction is attributed to the high concentrations of holes in A-ZnO and electrons in ZnO:Sn resulting in the dual diffusion of carries, as shown in the middle panel of Fig. 4d, which is similar as a traditional *p-n* homojunction. A depletion layer is therefore formed with a built-in electric field created by the space charge region. The bottom panel of Fig. 4d shows a typical energy diagram of the mimetic *p-n* homojunction of A-ZnO – *n*^+^-ZnO:Sn as well as the corresponding space charge at the boundary of microtube and film. It should be noted that the lack of hole-carriers and relatively low concentration of electron-carriers in undoped *n*-ZnO makes it impossible to form depletion layers with *n*^+^-ZnO:Sn or A-ZnO, as the top panel of Fig. 4d. Therefore, the diode-like behaviour cannot be observed in the undoped *n*-ZnO – *n*^+^-ZnO:Sn or A-ZnO – undoped *n*-ZnO homojunction structure, where a standard *n-n*^+^ homojunction behaviour is demonstrated as shown in Fig. 4c 46.

### Tuneable quench-free visible band emission in a single undoped A-ZnO microtube

**Figure 5:**
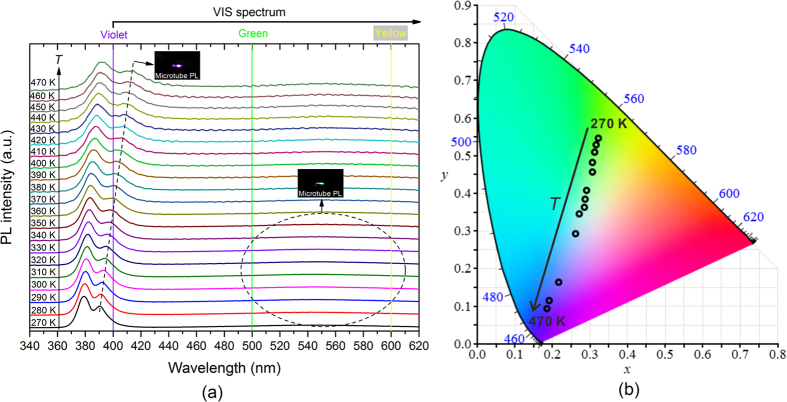
Temperature-dependent luminescence of an undoped A-ZnO microtube at high temperatures. (**a**) Temperature-dependent PL spectra of a single undoped A-ZnO microtube at 270–470 K and corresponding luminescence at various temperatures (insets). (**b**) The temperature-dependent luminescent colour located in the CIE colour space chromaticity diagram.

In addition to rich hole-carriers, novel temperature-dependent PL emission in visible band was observed from an undoped A-ZnO microtube, for the first time, due to the DAP transition. The PL spectra of a single ZnO microtube at various temperatures, as shown in Fig. 5a, illustrates the emission peak of DAP transition shifting fast from 390 nm to 414 nm with the temperature elevating from 270 K–470 K. The visible light emission is therefore from green (dominated by native donor defects with deep energy levels^47^) to violet (dominated by DAP transition). Considering the element components of the undoped ZnO microtube, the root of temperature-dependent light emission is attributed to the V_Zn_-related acceptors rather than substitution acceptor complexes, *e.g.* Sb_Zn_-2 V_Zn_^12^,^48^,^49^, As_Zn_-2 V_Zn_^13^,^49^, N_Zn_-V_O_^50^, *etc.* The DAP emission from substitution acceptor complexes is generally quenched by thermal ionisation at high temperature. However, V_Zn_-related DAP emission demonstrates high thermal stability ranging from 30 K to 470 K, as Figs 3a and 5a. Compared with the near band-edge transition (*i.e.* degeneration of FA, A^0^X and FX), the wavelength of DAP emission is more sensitive to temperature and locates in visible band with temperature elevating. Combining the temperature-dependent violet colour from DAP transition with the green colour from deep native defects transition, the visible luminescent colour of the undoped A-ZnO microtube can therefore be modulated in a large region of colour space, as shown in Fig. 5b, from green (0.33, 0.54) at 270 K to violet (0.19, 0.10) at 470 K across near white light (0.33, 0.33) at ~350 K. The luminescent property makes the undoped A-ZnO candidates for novel colour-tuneable light sources and thermosensors.

In this work, reliable free-standing undoped acceptor-rich ZnO (A-ZnO) microtubes were efficiently grown along [0001] direction by OVSP. The A-ZnO microtubes exhibited long lifetimes (longer than 1 year) against compensation/lattice-relaxation caused acceptor instability. The V_Zn_ were validated to be the stable shallow acceptors with binding energy of ~127 meV. The undoped A-ZnO microtube demonstrated low resistivity of 2.31 × 10^−2 ^Ω·cm and possibility for a mimetic *p-n* homojunction diode with *n*^+^-ZnO due to the high concentration of hole-carriers. In addition, quenching-free DAP emission from 390 nm to 414 nm with temperature increasing from 270 K to 470 K was found, by which the visible band emission from the A-ZnO can be modulated in a large region of colour space including white light. The present work provides a platform for fabrication of reliable ZnO with rich and stable acceptors. Most importantly, it opens up new opportunities for use of A-ZnO instead of *p*-ZnO in electronic and optical device applications, *e.g.* homojunction diodes, multi-wavelength light sources, thermosensors, *etc*.

## Methods

### Fabrication of ZnO microtubes

The ZnO ceramic precursor rod was synthesised by 99.99% ZnO powders in solid-state reaction. The powders were first pressed to a rod by isostatic pressing with 70 MPa and then sintered at 1200 °C for 12 h. The dimensions of sintered precursor rod were typically 6 mm in diameter and 50 mm in length. The homogenised optical heating process was performed in an optical floating zone furnace (10000H–HR-I-VPO-PC manufactured by Crystal Systems Co., Ltd.) equipped with four halogen lamps. The power of each lamp was set as 1000 W in order to achieve ~2000 °C on the precursor rod positioned at the furnace chamber centre. The ceramic precursor rod was rotated with 10 rpm during A-ZnO microtubes growing and meanwhile, carrier gas (Air/O_2_) was pumped into the chamber with 2 L/min. The growing process of microtubes generally finished within 100 mins.

### SEM and TEM analysis

The microtubes were directly placed onto a conductive tape for morphology examination by SEM (Hitachi S-3400N). The microtubes prepared for HRTEM characterisation were first dispersed by ethanol and dropped onto the TEM grid. Then the microstructures were captured by the TEM (JEOL-2010F) with an operating voltage of 200 KV.

### Raman spectrum analysis

The Raman scattering signals were captured by a high-resolution confocal Raman spectrometer (Horiba/Jobin-Yvon T64000) with an argon-ion laser of 514.5 nm as the excitation source. A 100× confocal objective with *NA* = 0.95 focused the raw laser beam to around 1 μm on the horizontally placed ZnO microtube facet (*i.e.* the *c*-axis of microtube perpendicular to the incident light). The scattered light was collected by the same objective under the backscattering configuration with a direct single stage of 640-mm focal length and an 1800 lines/mm grating for wavelength separation. A wide spectral range with the resolution of 1 cm^−1^ in Raman shift can therefore be detected by a liquid-nitrogen cooled CCD. The integration time of signal acquisition was set to 10 s.

### Temperature-dependent PL spectrum analysis

The PL excitation source was a 325-nm He-Cd fibre-coupled laser (Kimmon Koha IK3301R-G). The raw laser beam was focused by a 14× objective with *NA* = 0.5 onto the facet of an A-ZnO microtube. The backscattering PL was then collected by the same objective and filtered by a 1200 lines/mm grating for wavelength separation. A spectrograph with 750-mm focal length (Priceton Instruments Acton SP2750 at 300–900 nm spectral range) was employed to acquire the PL spectrum with the resolution of 0.023 nm. The microtube was horizontally placed onto a plate-heater in a vacuum chamber, in which the temperature can be controlled from 10 K to 90 K by liquid helium and 90 K–470 K by liquid nitrogen.

### XRD and XPS analysis

The microtubes were grounded to powder for XRD and XPS analyses. In addition, a standard undoped *n*-type ZnO substrate was employed as the control sample for the XPS analysis. The XRD used Cu-Kα1 (λ = 1.5418 Å) radiation generated at 36 KV/20 mA at a scanning speed of 0.02° min^−1^ in the range of 20°–80°. The XRD date was indexed by hexagonal wurtzite-type ZnO (JCPDS No. 80-0074). The XPS data were acquired by an AXIS-Ultra instrument from Kratos Analytical with monochromatic Al Kα radiation (225 W, 15 mA, and 15 KV) and low-energy electron flooding for charge compensation. In order to compensate to surface charges, binding energies were calibrated using C_1s_ hydrocarbon peak at 284.80 eV.

### Sample preparation for *I-V* characteristics and resistivity calculation for a single A-ZnO microtube

A single microtube was selected and placed onto a silica substrate. Both ends of the microtube were deposited by In/Ga electrodes and then the *I-V* curve of the microtube was acquired by 4200-SCS Semiconductor Characterization System. The electrical resistivity of the single microtube was calculated by


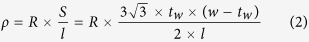


where *R* is the microtube resistance of ~2.27 KΩ obtained from the *I-V* curve, *l* is the microtube length of ~5 mm, and *S* is the microtube cross-sectional area of ~5.09 × 10^−8 ^cm^2^ based on the dimensions of ~100 μm in diameter with wall thickness of ~2 μm. For the A-ZnO-based homojunction, a microtube was first placed onto a silica substrate. Then a mask was used to partially deposit *n*^+^-ZnO:Sn film onto the microtube by radio frequency (RF) sputtering from a high purity ceramic target (SiO_2_:ZnO:SnO_2_, 6.5:51.43:42.07 at%) at room temperature. The mixed Ar/O_2_ gas of 40:1 were introduced into the chamber with a pressure of 1.4 Pa. The sputtering power was set as 80 W for 2 hours. The film thickness was ~1.5 μm. Then, In/Ga electrodes were deposited onto the end of microtube and the surface of film, respectively, as the inset in Fig. 4c. Finally, the *I-V* curve of A-ZnO-based homojunction were acquired by 4200-SCS Semiconductor Characterization System. The same procedure was also performed to prepare undoped *n*-ZnO film on an A-ZnO microtube and *n*^+^-ZnO:Sn film on undoped *n*-ZnO bulk for control experiments.

### Calculation of chromaticity values by PL spectrum

The temperature-dependent spectral power distributions, *I*(*λ, T*), from 380 nm to 780 nm were first obtained from the corresponding PL spectra. Then the tristimulus values for a colour can be calculated by


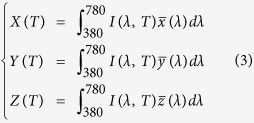


where 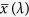, 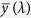 and 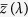 are CIE’s colour matching functions. The temperature-dependent chromaticity values (*x, y*) in the CIE colour space were therefore derived by


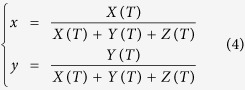


### Simulation method

The numerical simulation of electric field was performed by a finite element method (FEM) algorithm using COMSOL Multiphysics (licensed by COMSOL Co., Ltd.). A 2D cross-section model was developed in order to understand the light focusing by a ZnO hexagonal microrod (where *n* ≈ 2). The background was set as air (where *n* = 1). The perfect matching layers were applied as the open boundary conditions. A plane wave with the central wavelength of halogen lamp (*i.e.* 1080 nm) was stimulated as the incident light into the ZnO hexagonal microrod. The electric field distribution inside the microrod can therefore be calculated as shown in the middle panel of Fig. 1b.

## Additional Information

**How to cite this article**: Wang, Q. *et al.* Free-Standing Undoped ZnO Microtubes with Rich and Stable Shallow Acceptors. *Sci. Rep.* **6**, 27341; doi: 10.1038/srep27341 (2016).
